# Transition from ICU to home care with long-term invasive ventilation using a single-limb BiPAP circuit

**DOI:** 10.2478/jccm-2026-0004

**Published:** 2026-01-30

**Authors:** Mircea Stoian, Nicolae Demenciuc, Sergiu-Stefan Laszlo, Anca Motataianu, Dragoș-Florin Babă, Adina Stoian

**Affiliations:** Department of Anesthesiology and Intensive Care, George Emil Palade University of Medicine, Pharmacy, Science, and Technology of Targu Mures, Targu Mures, Romania; Pneumology Department, George Emil Palade University of Medicine, Pharmacy, Science, and Technology of Targu Mures, Targu Mures, Romania; Mures County Hospital, Targu Mures, Romania; Department of Neurology, George Emil Palade University of Medicine, Pharmacy, Science, and Technology of Targu Mures, Targu Mures, Romania; Emergency Institute for Cardiovascular Diseases and Transplantation, Targu Mures, Romania; Department of Pathophysiology, George Emil Palade University of Medicine, Pharmacy, Science, and Technology of Targu Mures, Targu Mures, Romania

**Keywords:** amyotrophic lateral sclerosis, non-invasive ventilation, invasive mechanical ventilation, mechanical insufflation-exsufflation device, respiratory failure, home mechanical ventilation, respiratory muscles

## Abstract

**Background:**

Patients with chronic respiratory failure caused by severe neuromuscular impairment often require long-term respiratory support. Invasive mechanical ventilation (IMV) via tracheostomy is usually provided in intensive care units (ICUs), but in carefully selected cases, it can be safely transitioned to home care. The use of a single-limb ventilator circuit (Single BiPAP circuit with Whisper Swivel II), intended initially for non-invasive ventilation (NIV), may represent a cost-effective and practical alternative for long-term home IMV.

**Case presentation:**

We present a 50-year-old male with progressive neuromuscular disease and chronic respiratory failure, who required long-term IMV through a tracheostomy tube. After stabilization in the ICU, ventilation was maintained at home using a Single BiPAP circuit with Whisper Swivel II, combined with a mechanical insufflation-exsufflation (MIE) device for airway secretion clearance. The patient’s family received structured training in tracheostomy care, ventilator operation, and secretion management. Over 32-month period, the patient maintained stable respiratory function, experienced a marked reduction in infectious exacerbations, and preserved an acceptable quality of life.

**Conclusion:**

In selected patients, long-term home IMV using a single-limb ventilator combined with an MIE device can be a safe, effective, and cost-efficient alternative to conventional ICU-based ventilation. Successful outcomes require structured patient and caregiver training, close follow-up, and coordinated multidisciplinary support.

## Introduction

Long-term invasive mechanical ventilation (IMV) via tracheostomy is generally reserved for intensive care units (ICUs) and specialized facilities, particularly in patients with severe neuromuscular disorders and chronic respiratory failure **[1]**. Amyotrophic lateral sclerosis (ALS) is a progressive muscle weakness, including respiratory muscle involvement, which ultimately results in respiratory failure **[2]**.

**Fig. 1.**
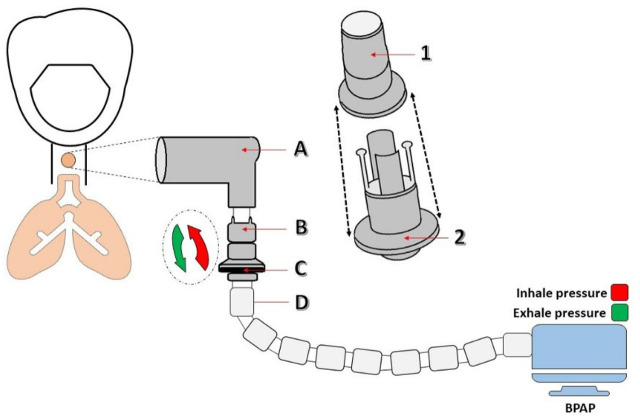
Single BiPAP circuit with Whisper Swivel II. A – Rotating connector; B – Valve, Whisper Swivel II; C – Exhaled air; D – Flexible tubing

In recent years, advances in ventilator technology have allowed the use of single-limb systems, such as the Single BiPAP circuit with Whisper Swivel II, originally designed for non-invasive ventilation (NIV), in invasive home ventilation **[3]**. Compared to traditional double-circuit ventilators used for tracheostomy ventilation, these devices are lighter, easier to operate, and more cost-efficient, with simplified exhalation ports and leak-compensation algorithms suitable for home care settin**gs** (Figure 1) [4].

Despite these technical advantages, there are limited reports describing prolonged use of such systems for invasive ventilation at home, particularly over periods exceeding two years, and even fewer cases in which care was provided exclusively by trained family members **[5]**. This case report presents the transition of a patient from ICU-based IMV to long-term home ventilation using a single-limb BiPAP circuit, combined with a mechanical insufflation-exsufflation (MIE) device for airway secretion clearance, highlighting both technical and caregiving aspects of this approach.

## Case report

A 50-year-old male with a two-year history of progressive lower-limb weakness (onset June 2020), later accompanied by bulbar involvement and declining vital capacity, presented with chronic respiratory failure secondary to progressive respiratory muscle weakness. Baseline neuroimaging (brain and cervical spine MRI) revealed no significant lesions, and electromyography demonstrated active and chronic denervation affecting cervical and lumbar territories, consistent with a progressive motor neuron disease. He was managed initially with riluzole. Over time, respiratory muscle weakness progressed with nocturnal apnea and ineffective cough, culminating in chronic ventilatory failure.

### ICU admission (December 2022)

The patient was initially evaluated in the emergency department with severe hypoxemic-hypercapnic respiratory failure, presented comatose, with shallow breathing (10/min), SpO_2_ 78%, and systemic instability. Emergent orotracheal intubation was performed in the emergency room, and IMV was initiated under continuous sedation with midazolam and fentanyl. He was subsequently admitted to the ICU for advanced respiratory and hemodynamic management. Chest computed tomography (CT) scan on day 1 showed right lower-lobe consolidation with small pleural effusion, involvement of the right middle lobe, and a centrilobular nodular pattern with perinodular ground-glass opacities; homogeneous ground-glass opacity was also evident in the left lower lobe (Supplementary Material - Figure S1). Laboratory testing showed leukocytosis, hypoglycemia, and elevated procalcitonin; arterial blood gases revealed severe metabolic acidosis (pH 7.088). Severity scores at admission were: SOFA 11, APACHE II 25, Ramsay 4, and CPIS 7 (See Supplementary Material - Table S1, for laboratory trends during hospitalization).

### Microbiology and anti-infective therapy

Bronchial aspirations, blood, and urine cultures were obtained. Empiric treatment (ceftazidime, levofloxacin, fluconazole) was started immediately. Respiratory cultures grew beta-hemolytic *Streptococcus* group G, *Achromobacter dentrificans,* and *Candida albicans.* After 19 days, *Acinetobacter baumannii* was isolated, prompting a switch to imipenem 4x500 mg for 8 days, followed by de-escalation to ampicillin/sulbactam for 11 days (See antimicrobial timeline in Supplementary Material - Figure S2).

### Airway and ventilatory strategy

Extubation to NIV was not successful because of severe respiratory muscle fatigue and ineffective cough. On ICU Day 9, a percutaneous tracheostomy with a cuffed cannula was performed, and IMV continued. Despite clinical improvement, repeated trials of supported modes failed due to CO_2_ retention and weakness; the team chose long-term invasive ventilation via a single-limb BiPAP circuit with Whisper Swivel II connected to the tracheostomy ventilators. This single-limb system uses an integrated leak port for passive exhalation and includes leak-compensation algorithms, while being lighter and easier to operate in a home-care setting.

### Bronchoscopy reassessment and current status

**Fig. 2.**
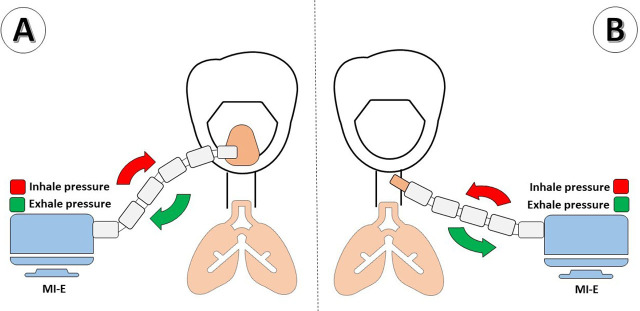
Mechanical Insufflation-Exsufflation Device. A - The MIE applies positive pressure through a mask; B. The MIE applies positive pressure through the tracheostomy connector.

At 7 months after ICU discharge (August 23, 2023), bronchoscopy showed a clean tracheobronchial tree with only minimal focal inflammation at the right upper-lobe orifice (Figure 2). Nutritional needs were met with semi-fluid enteral intake and oral supplements; PEG placement was discussed but deferred. At the most recent follow-up (age 53), the patient remained ventilator-dependent but clinically stable, with preserved communication and acceptable quality of life under family-led care and periodic specialist-scheduled reassessment. Throughout 2025, he remained free of hospital readmission and underwent three elective tracheostomy cannula changes during routine subsequent evaluation visits.

### Discharge planning caregiver training

After a 44-day ICU stay, the patient was alert, cooperative, hemodynamically and respiratory stable, and expressed a strong preference for home care rather than transfer to a long-term facility. A structured training program was completed with the primary caregiver (his wife), covering tracheostomy care, ventilator setup and troubleshooting, secretion suctioning, hygiene of the stoma, recognition of respiratory decompensation, and emergency procedures. The home environment was equipped with suction, spare circuits, filters, and contingency power solutions. Scheduled reevaluations were arranged at 3–4-month intervals, including cannula replacements and ventilator parameter optimization.

**Fig. 3.**
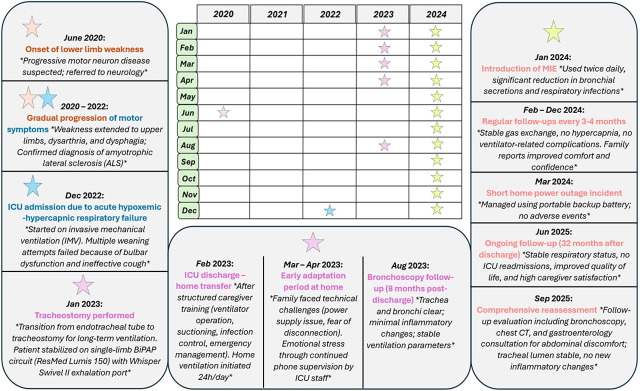
Timeline of the patient’s clinical course. Sequence of major clinical events and interventions from ICU admission to long-term home invasive ventilation and 32-month follow-up using a single-limb BiPAP circuit and mechanical insufflation-exsufflation (MIE).

A chronological overview of the patient’s clinical course, including ICU admission, tracheostomy, transition to home invasive ventilation and scheduled follow-ups, is presented in Figure 3.

### Home course and airway clearance

At home, the patient experienced intermittent infectious exacerbations triggered by secretion from expiratory muscle weakness, necessitating transient ventilator adjustments and short antibiotic courses guided by previous microbiology and labs (Supplementary Material - Tables S2–S3). From May to November 2023, no respiratory infectious exacerbations were recorded. In June 2023, given airway colonization with a multidrug-resistant organism and aligned with macrolide stewardship guidance, prophylactic azithromycin 500 mg three times weekly was initiated.

**Fig. 4.**
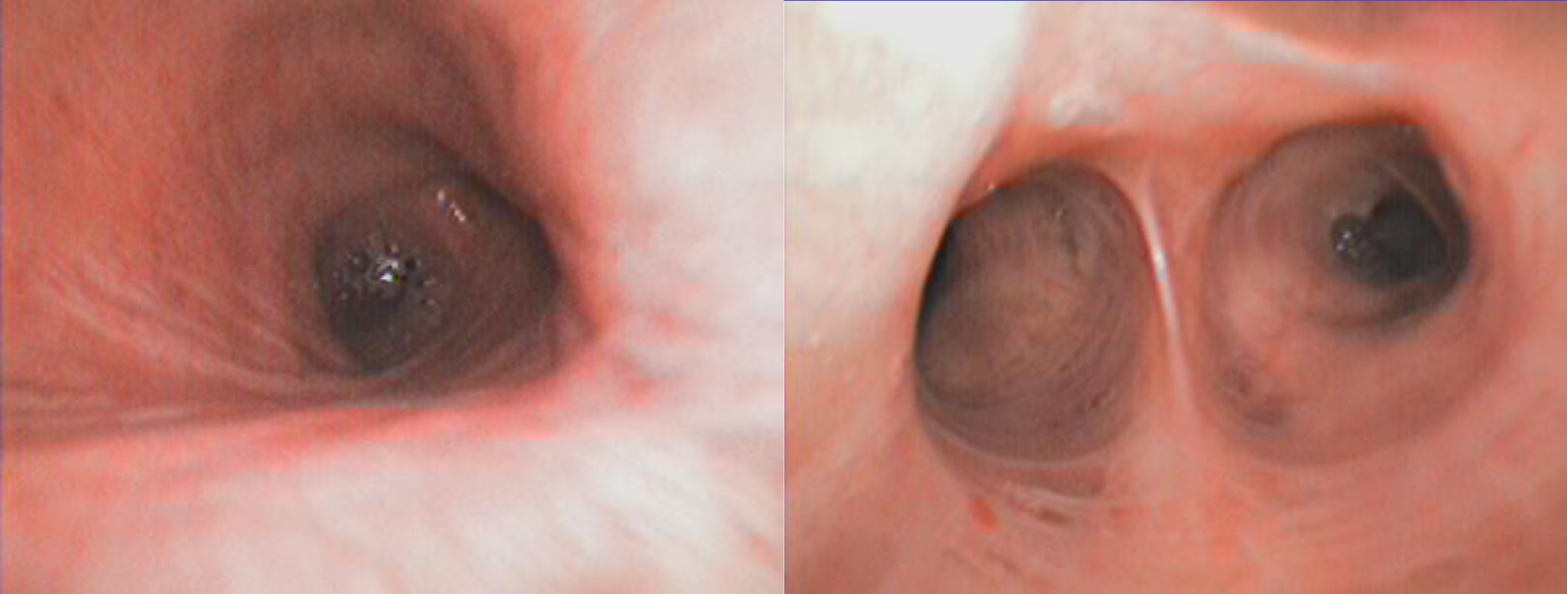
Bronchoscopy. Bronchoscopy performed on August 23, 2023, showing a clean trachea and bronchi with minimal inflammatory changes at the emergence of the right upper lobe bronchus (8 months after discharge).

To augment cough effectiveness and reduce secretion burden, a MIE device was introduced in January 2024 and connected via the tracheostomy (Figure 4). The device alternates positive and negative airway pressures to mobilize secretions toward the upper airway for subsequent suction. Following MIE implementation, infectious relapses ceased over the subsequent 12 months.

### Patient and Caregiver Perspectives

Although formal quality-of-life tools were not used, both the patient and his wife reported maintained comfort, stable communication, and improved emotional well-being after returning home. Early challenges included high maintenance costs, brief structured caregiver training, and the need for close cooperation with the ICU team. This qualitative feedback enhances objective clinical data and offers valuable insights into the psychosocial aspects of long-term home ventilation.

In the figure, the patient’s disease progression, ICU admission, tracheostomy, transition to home ventilation, MIE initiation, and long-term follow-up milestone at 32 months are presented.

## Discussion

Long-term home mechanical ventilation (HMV) is increasingly used in the management of patients with advanced neuromuscular disorders such as ALS once respiratory muscle weakness leads to chronic respiratory failure. Traditionally, IMV via tracheostomy in these patients is maintained with dual-limb ICU-grade ventilators in specialized facilities [6,7,8]. The present case illustrates the feasibility of transitioning a tracheostomized ALS patient from ICU-based IMV to long-term home care using a single-limb BiPAP circuit with Whisper Swivel II, supported by a MIE device, for an unusually long subsequent evaluation period of 32 months with family-led care.

Single-limb ventilator systems are typically designed for NIV and utilize a passive exhalation port to eliminate exhaled gases, reducing the complexity, weight, and cost compared to dual-limb ICU ventilators [9,10,11]. Their application in invasive ventilation for tracheostomized patients is less well-documented. In contrast to prior reports that mainly describe short-term use of single-limb ventilators to expedite ICU discharges [12,13,14], our case documents sustained home invasive ventilation for more than 32 months with a tracheostomy using a single-limb BiPAP circuit, highlighting long-term feasibility in a resource-limited setting. The sustained use of such systems for over two years in ALS patients, as presented here, has rarely been reported, underlining the novelty of this case.

Effective secretion clearance is a key determinant of survival and quality of life in ALS patients with IMV [15,16]. Impaired cough due to expiratory muscle weakness increases the risk of mucus plugging, atelectasis, and ventilator-associated pneumonia (VAP) [17]. MIE devices can significantly improve secretion mobilization, reduce infection rates, and decrease hospitalization needs [18,19]. In our patient, the initiation of MIE led to a complete cessation of respiratory infectious exacerbations over 12 months, in line with evidence from Chatwin et al. [18] and Vianello et al. [17]. Preventing infections in long-term ventilated patients is challenging, particularly in those with chronic airway colonization. Ventilator-associated pneumonia remains one of the most frequent and serious complications [20,21,22]. Recent findings by Stoian et al. (Antibiotics, 2025) emphasize the multifactorial etiology of VAP and highlight the importance of individualized antimicrobial strategies, strict hygiene protocols, and caregiver education in reducing incidence [23]. Our case demonstrates that rigorous caregiver training, combined with targeted antibiotic use and MIE integration, can minimize infectious burden even in high-risk patients. Similarly, Stoian et al. (Biomedicine, 2025) reported that risk factors for recurrent respiratory infections in long-term ventilated ICU patients include suboptimal secretin clearance, inadequate ventilator maintenance, and insufficient caregiver training [24]. All these risks were actively addressed in our patient through a structured discharge protocol, equipment optimization, and periodic reassessment. Nutritional status is another critical factor influencing the prognosis of ALS patients on long-term IMV [25,26,27]. Malnutrition and muscle wasting can accelerate respiratory decline and reduce survival. Stoian et al. (Nutrients, 2025) emphasize the importance of tailored nutritional interventions in ICU patients, including early recognition of dysphagia, adequate protein-energy intake, and micronutrient supplementation [28]. In our case, despite bulbar involvement, nutritional goals were achieved through a semi-fluid oral diet with supplementation, postponing the need for percutaneous endoscopic gastrostomy (PEG).

Comparing our approach to existing literature, home-based IMV in ALS is feasible but often limited by the availability of specialized home ventilation programs, caregiver willingness, and technical support [29,30,31]. In several series, survival with home IMV in ALS ranges from 10 to 30 months after initiation [32,33]. Our patient’s 32-month stability suggests that combining a simplified ventilator platform with proactive secretion management may extend this survival window, especially in resource-limited settings.

However, using single-limb circuits in invasive ventilation raises concerns. Problems like rebreathing risk, inadequate CO_2_ removal, and increased dead space have been reported. In our case, careful monitoring of end-tidal CO_2_ and regular ventilator checks ensured safe operation. During the first three months after discharge, we used a portable capnograph for intermittent end-tidal CO_2_ (EtCO_2_) checks—initially daily during the first two weeks, then weekly—complemented by arterial blood gases at scheduled follow-ups every three months. Ventilator logs and leak values were reviewed at each visit, and the Whisper Swivel II exhalation port and filters were inspected and replaced according to the manufacturer’s recommendations to prevent partial obstruction and excess dead space. No episodes of clinically relevant hypercapnia or rebreathing were detected, and PaCO_2_ remained within target ranges throughout follow-up (see Supplementary Material Table S2).

This case also has implications for healthcare systems, particularly in countries with limited access to long-term care facilities. Transitioning selected patients to home IMV can reduce ICU bed occupancy, lower costs, and improve patient autonomy [37,38]. Nevertheless, success depends on comprehensive caregiver training, availability of back-up equipment, and accessible multidisciplinary scheduled reassessment.

Several ventilator systems are available for long-term invasive home ventilation, including dual-limb ICU-grade ventilators, turbine-driven portable devices, and hybrid systems with internal leak compensation algorithms. Dual limb ventilators ensure precise tidal volume control and optimal CO_2_ clearance, but they are more expensive and require proper training, close supervision, and regular safety checks. In this context, using a single-limb BiPAP ventilator, like the one used for the presented patient, is an appropriate and recommended option for long-term invasive ventilation at home due to its favorable cost-effectiveness, ease of use, and stable performance in resource-limited conditions.

Limitations include the single-patient design, which precludes generalization, and the absence of formal cost-effectiveness analyses comparing single-limb systems. To enhance generalizability, future studies should include multicenter, prospective cohorts of tracheostomized patients managed with simplified single-limb ventilator systems, with predefined safety endpoints (e.g., hypercapnia, rebreathing) and health-economic analyses comparing home-based and facility-based invasive ventilation. Future research should evaluate long-term safety, cost savings, and quality-of-life outcomes in larger cohorts.

## Conclusion

This case demonstrates that long-term home invasive ventilation using a single-limb BiPAP circuit combined with mechanical insufflation-exsufflation can be a safe, effective, and cost-efficient alternative to conventional ICU-based ventilation in selected patients with neuromuscular disease. Successful outcomes require careful patient selection, structured caregiver training, regular subsequent evaluation, and multidisciplinary support.

## Abbreviations

ALS: Amyotrophic lateral sclerosis
BiPAP: bilevel positive airway pressure
BiPAP-ST: bilevel positive airway pressure in spontaneous/timed mode
COVID-19: Coronavirus disease 2019
CT: computed tomography
EB: base excess
EMG: electromyography
FVC: forced vital capacity
GGO: ground-glass opacity
HMV: home mechanical ventilation
ICU: intensive care unit
INR: international normalized ratio
IMV: invasive mechanical ventilation
MDR: multidrug-resistant
MIE: mechanical insufflation-exsufflation
MV: mechanical ventilation
NCS: nerve conduction studies
NIV: non-invasive ventilation
PCF: peak expiratory cough flow
SARS-CoV-2: severe acute respiratory syndrome coronavirus 2
SIMV: synchronized intermittent mandatory ventilation
TIV: tracheostomy invasive ventilation
WBC: white blood cells
